# Multidisciplinary strategies to treat painful mononeuropathies in the upper extremity: from lab to bedside

**DOI:** 10.1177/17531934241240389

**Published:** 2024-05-15

**Authors:** Mienke Rijsdijk, Sami Tuffaha, Henk Coert

**Affiliations:** 1 Pain Clinic, Department of Anaesthesiology, University Medical Centre Utrecht, Utrecht, the Netherlands; 2 Department of Plastic Surgery, Johns Hopkins Medical Centre, Baltimore, Maryland, USA; 3 Department of Plastic Surgery, University Medical Centre Utrecht, Utrecht, the Netherlands

**Keywords:** Multidisciplinary, pain, mononeuropathy, phenotyping

## Abstract

Neuropathic pain in the upper extremity is a serious problem, commonly involving relatively young patients. The pain causes loss of function and productivity, changes a patient’s lifestyle and can progress into a chronic pain syndrome with secondary psychosocial co-morbidities. Treating patients with a painful mononeuropathy remains challenging, with a monodisciplinary approach often having limited treatment efficacy. This narrative review discusses how to deal with this challenge in the treatment of patients with peripheral nerve injury pain, addressing the four important pillars: (1) diagnosing a painful mononeuropathy; (2) clinical pain phenotyping; (3) personalized pain treatment; and (4) using a multidisciplinary team approach.

## Background

Chronic pain affects approximately 20% of the world’s population and has an enormous socioeconomic and healthcare burden (Breivik et al., 2006; Gaskin and Richard, 2012; Patel et al., 2012; Phillips, 2006). It is more prevalent than diabetes, coronary heart disease and cancer combined (National Center for Health Statistics (US), 2006).

A common aetiology of chronic pain is peripheral nerve injury. All wounds, varying from small cuts to whole limb amputations, are accompanied by some degree of peripheral nerve injury. There are approximately 26.7/100,000 new cases of neuropathic pain caused by traumatic and iatrogenic (postoperative) nerve injuries every year (Dieleman et al., 2008). One in three injuries in the emergency department is a hand injury and 50% of all these hand injuries involve serious damage to digital nerves (van der Avoort et al., 2013). Neuropathic pain in the upper extremity is a serious problem, commonly involving relatively young patients. The pain causes loss of function and productivity, changes a patient’s lifestyle and can progress into a chronic pain syndrome, which can be severely disabling, sometimes also leading to secondary psychosocial co-morbidities, such as anxiety, depression and substance abuse (Breivik et al., 2006; Burger et al., 2007).

Painful mononeuropathies can result from a number of causes, including nerve injury, compression or entrapment. Non-surgical treatment of painful mononeuropathy includes analgesic oral or transdermal drug treatment, nerve blocks and neuromodulation (e.g. spinal cord of dorsal root ganglion [DRG] stimulation or transcutaneous electric nerve stimulation [TENS]). Surgical treatment may involve neurolysis, nerve repair, muscle burying, regenerative peripheral nerve interfaces (RPNI) and targeted muscle reinnervation (TMR). Treating patients with a painful mononeuropathy remains challenging because current interventions have limited efficacy. An example of such a treatment is opioid analgesics, with very limited efficacy in patients with neuropathic pain and causing severe side effects, such as addiction. The misuse and addiction to opioids prescribed for pain has led to the current opioid crisis in several countries (Wilson et al., 2020).

There are several reasons why treatment effect, either non-surgical or surgical, is so disappointing. Patients with peripheral nerve injury pain form a heterogeneous population regarding co-morbidities influencing recovery, e.g. diabetes, psychiatric symptoms but also regarding the pathophysiologic mechanism of pain resulting in different pain phenotypes known to respond differently to pain treatment.

How to deal with these challenges in the treatment of patients with peripheral nerve injury pain will be discussed in this narrative review, addressing the four important pillars: (1) diagnosing painful mononeuropathies; (2) clinical pain phenotyping; (3) personalized pain treatment; and (4) using a multidisciplinary team approach.

## Diagnosing painful mononeuropathies

**Figure 1.**
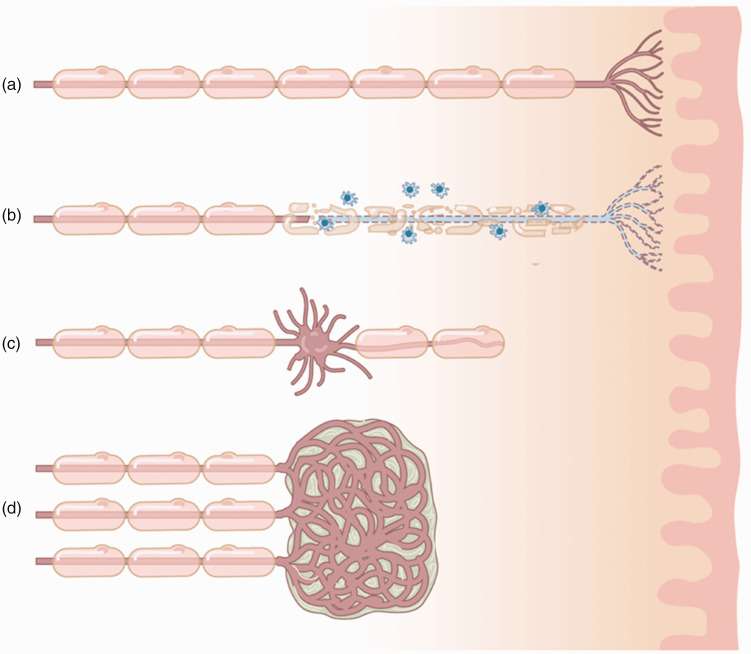
Peripheral nerve injury and neuroma formation. (a) Healthy myelinated peripheral nerve innervating the skin. (b) Peripheral inflammatory process damaging myelin and the nerve endings. (c) Partial nerve laceration or severe crush injury resulting in a neuroma-in-continuity and (d) severed nerve left in discontinuity resulting in an end-bulb neuroma comprised of a disorganized tangle of regenerating nerve fibres.

Upper extremity trauma often results in peripheral nerve injury, which in turn can lead to the formation of a neuroma when the axonal nerve fibres are damaged. When a nerve is severed and left in discontinuity or inadequately repaired, an end-bulb neuroma will form comprising a disorganized tangle of regenerating nerve fibres (Figure 1(d)). In addition, a partial nerve laceration or severe crush injury can result in a neuroma-in-continuity (Figure 1(c)). Many, but not all, neuromas result in neuropathic pain. The reported incidence of painful neuroma formation after upper extremity amputations is in the range of 4%–25% (Fisher and Boswick, 1983; Geraghty and Jones, 1996; van der Avoort et al., 2013). Specific symptoms that manifest from traumatic neuromas include tenderness, spontaneous pain, allodynia, hyperalgesia and mechanical or thermal hypersensitivity in the innervated area. Neuropathic pain after peripheral nerve injury remains a challenge for both patient and physician. We have previously proposed an algorithm to diagnose painful mononeuropathies based on a history of known or suspected nerve injury, pain with neuropathic features within the distribution of the affected nerve, combined with either a positive Tinel sign, positive response on a local anaesthetic block or confirmation of a neuroma of neuronal discontinuity with ultrasound or magnetic resonance imaging (Arnold et al., 2019).

The intensity and nature of the pain arising from a painful mononeuropathy may vary, depending on different pathophysiological mechanisms. It is essential to first understand these mechanisms before a pain treatment can be personalized for a patient with a painful mononeuropathy.

### Pathophysiology of painful mononeuropathy

Different types of pain arise secondary to a nerve injury, each with a distinct pathophysiologic basis. When a nerve is injured and not appropriately repaired, a neuroma will form consisting of a mass of regenerating, entangled axons, proliferating Schwann cells and inflammatory cells within a collagen matrix. The typical neuroma occurs after a complete nerve transection left in discontinuity, such as in an amputation. However, partial or complex nerve injuries can also produce neuromas. Only nerves that supply the skin can produce painful neuromas, whereas injured nerves that exclusively supply muscle do not. This is likely because cutaneous afferent fibres include a larger proportion of nociceptive A delta and C fibres. While 50%–80% of the axons that supply skeletal muscle are afferent, the large majority are proprioceptive rather than nociceptive. It is not clear why some cutaneous nerve injuries produce painful neuromas while others result in non-painful neuromas. The answer may be found in peripheral and/or central modulation of the nociceptive signal. Peripheral modulation includes ectopic impulse generation and peripheral sensitization leading to persistent nociceptive signals being transmitted to the central nervous system. For example, within the painful neuromas, sodium channels Nav1.3, 1.7 and 1.8, elongation factors associated with translation (EFT1/2) and mitogen-activated protein (MAP) kinase have been shown to be upregulated amplifying the pain signal (Black et al., 2008; Kretschmer et al., 2002). Furthermore, local inflammation has been heavily implicated in neuroma pain, with upregulation of pro-inflammatory cytokines such as TNF-α and downregulation of IL-10 in painful versus non-painful neuroma tissue samples (Held et al., 2019). However, there are also distinct downstream manifestations of neuropathic pain that differ with regard to their qualia as well as their underlying pathophysiologic mechanisms. Using their spared nerve injury model, Dorsi et al. (2008) demonstrated that neuropathic pain also arises from the process of collateral sprouting from intact cutaneous axons adjacent to the region of skin denervated by the nerve injury. This explains why patients with nerve injuries will often describe dysesthesias in the region of skin that would be expected to be insensate and painless.

All these peripheral events may result in increased afferent input in the DRG and spinal cord dorsal horn. The increased afferent input in the DRG and spinal cord dorsal horn can induce central neuroplasticity, triggering alterations in the spinal cord and brain. This neuroplasticity contributes to heightened pain sensitivity, changes in sensory information processing and the onset of persistent pain states, commonly known as central sensitization. Within the spinal cord and brain stem, central sensitization serves as a pivotal driver and amplifier of the aforementioned pain processes. Moreover, it may give rise to distinct pain phenotypes in a subset of individuals with nerve injuries. Maladaptive transformations in the central nervous system involve synaptic plasticity, adjustments in neurotransmitter release and modifications in the connectivity of neural circuits associated with pain. Central sensitization, coupled with inadequate cortical reorganization, is believed to underlie phantom limb pain in amputees and the emergence of generalized pain extending beyond the anatomical region supplied by the affected nerve(s). Phantom pain occurs more often with digital nerve and radial sensory nerve neuromas than neuromas occurring in the proximal upper extremity, perhaps due to greater cortical representation in the homunculus. Also suggesting central determinants, children are known to be less at risk for experiencing pain arising from a neuroma, presumably because they possess greater central plasticity allowing for modulation and silencing of the dysregulated peripheral signals.

Finally, decreased central pain inhibitory pathways often in correlation with affective disorders influence pain intensity and phenotype. It is therefore important to know if these pathophysiological processes are playing a role in the pain the patient you are treating to be able to offer a personalized pain treatment.

## Clinical pain phenotyping

The variety in the extent and type of nerve damage influences individual somatosensory profiles, a mosaic of hyperalgesia, hyperaesthesia, allodynia, and hypoaesthesia and hypoalgesia. Hyperalgesia and hyperaesthesia denote heightened sensitivity, with hyperalgesia specifically referring to an increased sensitivity to painful stimuli. Allodynia involves the perception of pain from typically non-painful stimuli. On the other hand, hypoaesthesia and hypoalgesia indicate reduced sensitivity, with hypoaesthesia encompassing decreased sensitivity to various sensory inputs and hypoalgesia representing a decreased sensitivity to painful stimuli specifically. These terms are crucial in describing alterations in sensory perception (Table 1).

**Table 1.** Alterations in sensory perception.

Term	Definition	Characteristics
Hyperalgesia	Increased sensitivity to painful stimuli, resulting in an exaggerated response to noxious sensations	Perception of pain is intensified
Hyperaesthesia	Heightened sensitivity to various sensory stimuli, not limited to pain	Increased response to touch, temperature and pressure
Allodynia	Perception of pain in response to normally non-painful stimuli	Non-painful stimuli (e.g. light touch) cause pain
Hypoaesthesia	Reduced sensitivity to sensory stimuli, leading to decreased perception of touch or pressure	Diminished ability to feel and interpret sensory information
Hypoalgesia	Decreased sensitivity to painful stimuli	Reduced perception of pain, making individuals less responsive to noxious stimuli

Clinical pain phenotyping is based on these alterations in sensory perception and is an important step in unravelling whether the nature and intensity of the pain are modulated by central processes such central sensitization, decreased central pain inhibitory pathways and psychosocial factors, or whether the pain is mainly caused by peripheral ectopic activity from within the neuroma. Diagnostic tools assessing these factors aiding in the phenotyping of pain are quantitative sensory testing (QST), conditioned pain modulation (CPM), diagnostic peripheral nerve blocks and psychometric questionnaires.

### Quantitative sensory testing

QST is a psychophysical test used to quantify somatosensory sensation under normal or pathological conditions (Vollert et al., 2016). Employing standardized stimuli, such as thermal, mechanical and electrical modalities, QST measures various sensory thresholds, including detection and pain thresholds, providing numerical data rather than relying solely on subjective reports. This method enables the evaluation of tactile, thermal and pressure sensations, aiding in the identification of abnormalities and changes in sensory functions. With QST, the clinician can assess if there is central modulation of peripheral neuronal activity resulting in increased pain intensity or altered pain sensation. There are indications that QST allows for detecting three subsets of patients with neuropathic pain who may display specific phenotypes (that may overlap): sensory loss (31%); mechanical hyperalgesia (63%); or thermal hyperalgesia (46%) (Baron et al., 2017). It is likely that different underlying mechanisms are responsible for the generation and maintenance of the pain in these subsets. Multiple studies indicate that subsets of patients identified with QST may respond differently to pain treatment (Bouhassira and Attal, 2023).

### Conditioned pain modulation

CPM can be assessed with cuff algometry, a process that involves the modulation of pain perception through the application of a conditioning stimulus at a remote site (Cummins et al., 2020; Graven‐Nielsen et al., 2017). In cuff algometry, a blood pressure cuff is typically placed around a limb, acting as the conditioning stimulus. The cuff is inflated to induce ischemic pain, serving as the conditioning pain stimulus. Simultaneously, the individual may undergo a test stimulus, such as pressure or heat, applied at a different site. The assessment involves measuring the change in pain perception during the test stimulus while the conditioning stimulus is applied. This method allows researchers and clinicians to evaluate the effectiveness of endogenous pain modulation systems: the ability of the patient’s brain to reduce the experienced nociceptive intensity when a second nociceptive stimulus is offered. CPM is also referred to as descending inhibitory control. Tricyclic antidepressant drugs are able to improve this central inhibitory control of pain, thereby reducing pain intensity (Bannister and Dickenson, 2017; Hiroki et al., 2017).

### Diagnostic nerve blocks

By performing diagnostic nerve blocks in patients diagnosed with a painful mononeuropathy, transduction of the nociceptive signal in the peripheral nerve is temporarily blocked. If pain disappears completely, it is hypothesized that mainly peripheral ectopic activity is causing the pain. If the pain does not disappear, but there is numbness in the innervated area of the skin of the target peripheral nerve, confirming a correct technical nerve blockade, it is hypothesized that central processes also play a role and surgery on the peripheral nerve may be less efficacious (Stokvis et al., 2010b). To be able to carefully assess the outcome of the peripheral blocks, we have developed a clinical protocol performing three separate peripheral nerve blocks with either saline, lidocaine 2% or bupivacaine 0.25%. The patient will receive the three drugs on separate days but is blinded to the order in which they are administrated and is asked 1 h after administering the drug if the block reduced the pain and for how long. We believe it is important to follow this protocol to minimize placebo effects and socially desirable answers manipulating the outcome. In previous studies by our team, we show that these blocks are predictive for surgical success (Oomen et al., 2014; Stokvis et al., 2010a, 2010b).

### Psychometric questionnaires

There is mounting evidence that the efficacy of pain treatment, whether conservative or surgical, is influenced by psychosocial factors. Psychological factors include catastrophizing thoughts, anxiety and depressive symptoms, and coping malfunctioning. Catastrophizing has been identified as an important modifier of treatment effect and is a known predictor of chronic pain development after surgery (Glare et al., 2019; Kehlet et al., 2006). Depressive symptoms are known to maintain a pain state and have a bidirectional relation with chronic pain (Edwards et al., 2016). This means that depressive symptoms may make the patient susceptible for chronic pain development and chronic pain may lead to secondary depressive symptoms. In recent studies, the combination of psychological symptoms has been assessed, identifying subgroups within the heterogenous pain patient population with low, intermediate and high psychological burden influencing treatment outcome (Bäckryd et al., 2018; Gerdle et al., 2019; Larsson et al., 2017).

An important social factor is the presence of a personal injury litigation, employment status or Workman’s compensation. Within the compensation process, it is not in the financial interest of the injured patient to recover, greatly influencing treatment outcomes (Collie et al., 2019).

In addition, in neuroma patients, employment status and lifestyle factors such as smoking were significantly related to worse outcome, with a relative risk of 2.10 (Stokvis et al., 2010a).

In summary, it is important to have a working understanding of the biopsychosocial factors playing a role in the experienced pain intensity, quality of life and prognosis, making a mechanism based and personalized treatment regimen possible.

## Personalized pain treatment

Depending on the results from the additional pain diagnostic tools, a personalized treatment regimen can be proposed. First, it is important to acknowledge factors that will undermine treatment efficacy such as a compensation lawsuit, clinically significant anxiety disorder or depression. These need to be addressed first before advancing into analgesic interventions (e.g. surgery, neuromodulation). Second, when central sensitization processes have come into play and/or there is an impaired conditioned pain modulation, conservative treatment options (e.g. analgesic drugs) may be initiated first. Ketamine and lidocaine infusions and tricyclic antidepressant drugs are known to reduce central sensitization processes and enhance central descending inhibitory control of pain (Challapalli et al., 2005; Cohen et al., 2018; Fitzcharles et al., 2021; Hiroki et al., 2017). Patients showing no psychosocial co-morbidities or central pain modulation are ideal candidates for interventional pain treatment by pain specialists and/or peripheral nerve surgeons.

### Interventional pain treatment by pain specialists

The arsenal of interventional pain treatments by pain specialists consists of nerve blocks using (pulsed) radiofrequency ([p]RF) or cryotherapy, and neuromodulation (spinal cord stimulation, DRG stimulation or transcutaneous electric nerve stimulation [TENS]). The efficacy of these treatments varies depending on pain duration, phenotypes and pain-related co-morbidities.

#### Nerve block including PRF treatment and cryotherapy

During PRF, a high-voltage electric field is created around a nerve or DRG. It is hypothesized that PRF has membrane-stabilizing and anti-inflammatory effects leading to its analgesic properties (Sam et al., 2021). There is substantial evidence that PRF is effective for radicular pain, post-herpetic neuralgia and occipital neuralgia (Chang, 2018). Unfortunately, there are insufficient data for PRF treatment for neuropathic pain of the upper extremity. Ultrasound-guided percutaneous cryoneurolysis has been used in clinical practice for decades to treat post-amputation pain, but did not reduce phantom limb pain 4 months after treatment in a recent randomized controlled trial (RCT) (Ilfeld et al., 2023). There is no trial assessing stump pain after cryoneurolysis.

#### Neuromodulation

Neuromodulation works through the principle of stimulating fast–velocity mechanoreceptive Aβ fibres to prevent slower moving nociceptive signals transmitted by Aδ and C fibres from reaching higher centres of the brain, resulting in analgesia. This has also been referred to as the gate control theory. Neurostimulation can be performed transcutaneous (TENS), at the site of the peripheral nerve, the DRG, the dorsal horn of the spinal cord and the brain. For TENS, there is low-quality evidence that it is superior to sham or no treatment for neuropathic pain (Knotkova et al., 2021). Peripheral nerve stimulation technologies have evolved rapidly, with devices available nowadays that can be placed percutaneously. There is low- to moderate-quality evidence that peripheral nerve stimulation is effective for neuropathic pain in an extremity (Deer et al., 2016; Knotkova et al., 2021; Xu et al., 2021). In a prospective, randomized, double-blind, crossover study, the efficacy and safety of a wireless neurostimulation device (StimRouter) was assessed in patients with severe intractable chronic pain (>3 months) of peripheral nerve origin after trauma or surgery (Deer et al., 2016). In the treatment group, 38% responded to treatment (with at least 30% reduction of pain) compared to 10% in the control group at the 3-month follow-up. No serious adverse events were reported throughout the trial and with a follow-up to 1 year. In another RCT (ICON study), patients with cluster headaches were effectively treated with occipital nerve stimulation resulting in fewer headache attacks (from 16.2 to 4.2 attack per week at the 1-year follow-up) without serious adverse events (Brandt et al., 2023).

With DRG stimulation, the neuronal cell bodies of the peripheral sensory neurons are directly stimulated (Esposito et al., 2019). For both DRG stimulation and spinal cord stimulation, high levels of patient satisfaction have been reported when these treatment modalities are used for chronic intractable pain (Hagedorn et al., 2022).

There is emerging evidence that besides the modulation of the somatosensory pathways, neurostimulation also influences the inflammatory response associated with chronic pain (Chakravarthy et al., 2019).

### Surgical options for neuroma-related pain

It is generally accepted that the most effective method to prevent painful neuroma formation is to restore continuity to the traumatized nerve at the time of injury. This can be accomplished with a primary epineurial repair in the setting of a sharp laceration or with nerve graft when a segmental defect is present. By restoring a pathway for the severed axons to regenerate towards their intended skin distribution, this presumably provides the greatest likelihood that they will find cutaneous sensory end organs to reinnervate such that they will turn off their regenerative machinery and become quiescent. Performing a nerve repair in the acute or subacute period is thought to decrease the risk of distal collateralization and centralization of pain that is more difficult to treat with peripheral interventions. For critical sensory and/or motor defects with a gap length that is amenable to grafting, we should strive to reconstruct the nerve to regain function if the distal nerve stump is available.

**Figure 2.**
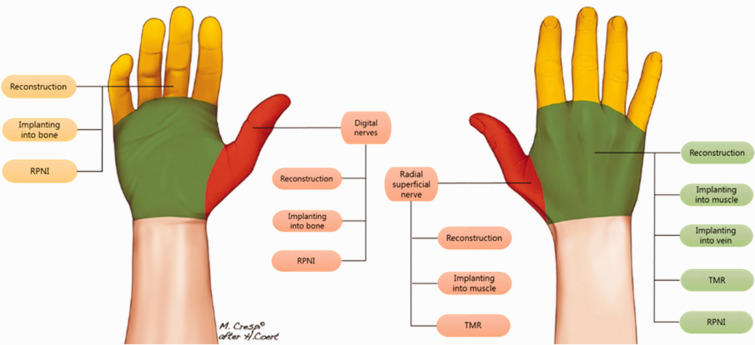
Left: palmar view of the hand, showing three surgical options for a painful neuroma at the level of the fingers: nerve reconstruction; nerve end burying in bone; and RPNI. Right: dorsal view of the hand and wrist showing surgical options for the radial sensory nerve: reconstruction; implanting in muscle; and TMR. Options for painful neuromas at level of the hand are also shown. RPNI: regenerative peripheral nerve interface; TMR: targeted muscle reinnervation.

However, in many scenarios, restoring continuity to the injured nerve is not possible or practical. For example, the distal nerve stump may not be available (i.e. amputation) or there may be an extensive zone of injury that would require an excessively long nerve graft (Figure 1(b)). In such cases, a neuroma will form at the terminal end of the nerve (Figure 1(d)). In the fingers, these neuromas have been shown to cause extreme pain in approximately 8% (van der Avoort et al., 2013). Burying the proximal stump of the injured nerve into nearby muscle (MB) or bone, either after neuroma resection or at the time of nerve injury/limb amputation, is one of the earliest described and most widely employed methods to treat and prevent symptomatic neuromas (Mackinnon et al., 1985). In Figure 2, we show different surgical options for painful neuromas at the level of the fingers, palm and wrist. Nerves can also be partially damaged causing an incomplete disruption of all axons. In these cases, a so-called neuroma-in-continuity can form, causing neuropathic pain (Figure 1(c)).

**Figure 3.**
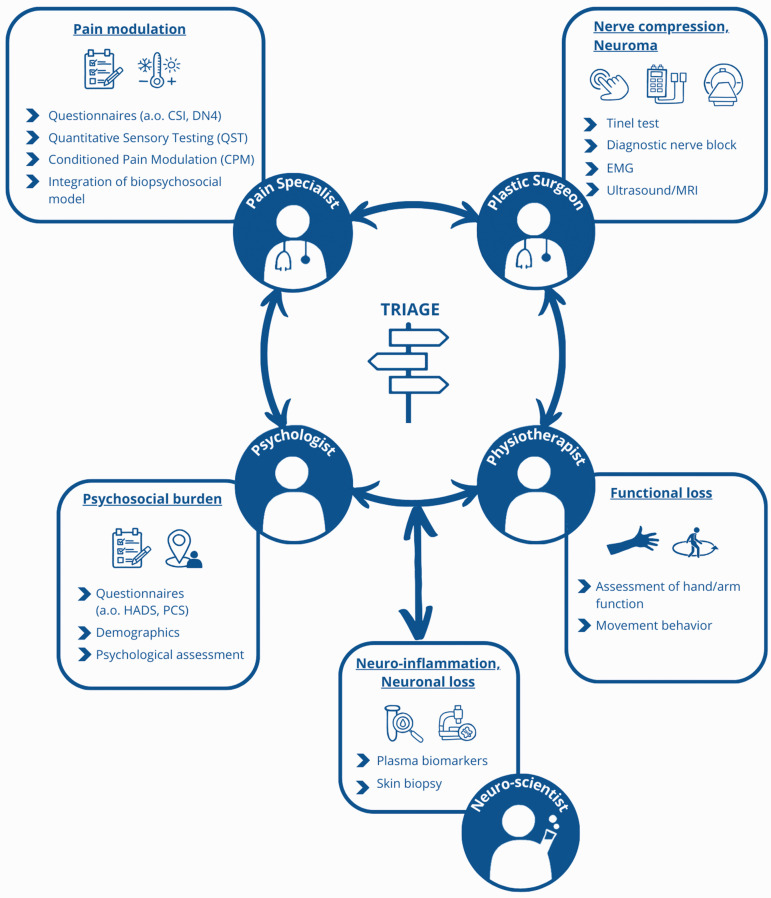
Proposed triage for a patient with suspected painful mononeuropathy involves referral to healthcare providers, such as a pain specialist, psychologist, physiotherapist and nerve surgeon, enabling a comprehensive assessment and appropriate redirection to other team members as needed. This collaborative approach ensures a thorough understanding of the patient’s condition and tailors interventions accordingly.

In the seminal study introducing the method performed in non-human primates, the morphological features of the neuroma that formed within muscle were qualitatively described as having well contained and less disorganized axonal growth. Importantly, this method does not prevent neuroma formation as muscle that is already innervated does not serve as a target for reinnervation by the axons regenerating from the injured nerve implanted within it.

More recently, with the advent of targeted muscle reinnervation (TMR) and regenerative peripheral nerve interface (RPNI) techniques, there has been a growing emphasis on providing denervated muscle as a target for reinnervation and thereby prevent neuroma formation altogether (Figure 2). The fundamental differences between TMR and RPNI are as follows: (1) TMR leverages a nerve-to-nerve coaptation to reinnervate the target muscle whereas RPNI involves direct neurotization with implantation of injured nerve stump within the substance of the muscle; and (2) the target muscle in TMR is a fully innervated muscle that resides in its native location whereas an RPNI is a non-vascularized muscle graft harvested from local or distant site.

The primary criticism of RPNI involves the need for muscle engraftment, which significantly limits the size of the RPNI that can be used. If the muscle graft is too large, central necrosis will occur, and even with a muscle graft small enough to avoid necrosis, significant fibrosis and resorption is expected. To address these limitations, we more recently introduced vascularized, denervated muscle targets (VDMT), which are essentially designer flaps fully raised on vascular leashes entering a nearby muscle (Tuffaha et al., 2020). Because the muscle flap is only attached by its vascular pedicle, complete denervation is ensured. We have also used this principle creating a distally based ‘flap’ of the muscle, which is vascularized, but denervated. The vascular leashes entering muscle are widely available and more abundant than the motor nerves required for TMR. While VDMT and RPNI rely solely on direct neurotization of the target muscle, TMR also seemingly relies to some extent on direct neurotization when the size-mismatched repair site is performed near or within the target muscle. The implications of direct neurotization versus nerve coaptation for neuroma prevention have yet to be adequately explored. Furthermore, the implications of having a smaller, engrafted muscle target (RPNI) versus a larger, vascularized muscle target (VDMT) have not been adequately defined. The most fundamental question underlying all these approaches involves the fate of regenerating nociceptive fibres entering a target muscle. Because pain fibres do not have a target sensory organelle to reinnervate, it remains unclear what the specific reinnervation ‘target’ is for this critical subtype of sensory axons that provides the signal that halts further regeneration. It is also unclear whether denervation of the target muscle is important in this regard. While some studies have demonstrated sensory reinnervation of muscle spindles and Golgi within muscle, none to our knowledge have specifically demonstrated pain fibres reinnervating these sensory organelles (Adidharma et al., 2022).

We recently found in a rodent model that both classic muscle burying (MB) and VDMT could innervate the target muscle; however, the traditional non-vascularized RPNI did not do this (unpublished data). This, in fact, questions the mechanism of action of the classic RPNIs in relation to MB and VDMT.

A practical downside to TMR is the relative paucity of usable motor nerves often necessitating additional exposure and incisions. In addition, the larger damaged nerve could be separated into different smaller bundles allowing for a better matching coaptation, thereby creating more intraneural scar and could potentially be a cause of persistent pain.

We should realize that sensory nerves often have overlapping areas of distribution. The radial sensory nerve and the lateral antebrachial cutaneous nerves in the distal forearm are examples of this. We found in anatomy studies that both nerves overlap considerably in their distribution and even both nerves intersect at different locations in the arm (Poublon et al., 2015). These anatomical variations should be considered when treating nerve pain due to damaged nerves. Diagnostic nerve blocks can be particularly helpful in such scenarios. In recent literature, no RCTs on this topic have been published yet. However, recent literature suggests that active (TMR, v-RPNI) treatment strategies for neuromas in the hand can be more successful than passive strategies. Anatomical location will dictate the strategy, as TMR, VDMT and muscle burying are not always possible, unless there is sufficient length of the digital nerves (Figure 2). RCTs are needed to provide a higher level of evidence for the preferred techniques for treatment of neuromas in the hand.

## A multidisciplinary team approach

At this time, unfortunately, treatment strategy is mainly dependent on the training background and individual biases of the doctor who sees the patient first. In most hospitals, patients are being treated by either pain management specialists or peripheral nerve surgeons separately. This has resulted in complete separate patient journeys, in which the treatment depends on the speciality of the involved physician rather than on the actual optimal strategy. Referral to the other group is usually only initiated if the current treatment regime has not been successful. This is not in the interest of patients. For example, some surgical interventions, like the denervation of the radial sensory nerve (RSN), are far more successful when performed within 12 months of nerve injury. For the RSN, in particular, it has been published that early denervation will yield pain reduction results of 70%–75%, which is good to excellent, versus treatment after 12 months, leading to pain reduction of only 10%–15%. In addition, patient factors, such as depression or catastrophic thinking, should be identified before treatment. In pain management teams, these factors are more integrated in the treatment algorithm than in surgical teams (Stokvis et al., 2010b).

All pain patients should have a comprehensive work-up using validated psychometric questionnaires, quantitative sensory and conditioned pain modulation tests, and patient-reported outcome measures. Using a common trunk intake for both pain management teams and peripheral nerve surgery teams would facilitate the comparison of outcomes. Even better, all pain patients should be seen in a multidisciplinary team consisting of at least pain specialists, psychologists, physiotherapists and nerve surgeons. In this setting, the different specialities will complement each other in terms of background and knowledge on pain and its psychosocial co-morbidities and functional loss. Patients will receive a personalized intake, work-up (e.g. nerve blocks, assessment of psychosocial co-morbidities, assessment of function and movement behaviour). Ideally, a psychiatrist, physical medicine and physicians, certified hand therapist, musculoskeletal radiologist and social health worker should be available when needed.
